# The Role of Age and Comorbidity Interactions in COVID-19 Mortality: Insights from Cardiac and Pulmonary Conditions

**DOI:** 10.3390/jcm13247510

**Published:** 2024-12-10

**Authors:** Raul Patrascu, Cristina Stefania Dumitru, Ruxandra Laza, Razvan Sebastian Besliu, Miruna Gug, Flavia Zara, Sorina Maria Denisa Laitin

**Affiliations:** 1 Department of Functional Sciences, “Victor Babes” University of Medicine and Pharmacy, 300041 Timisoara, Romania; patrascu.raul@umft.ro; 2 Department of Microscopic Morphology, “Victor Babes” University of Medicine and Pharmacy, 300041 Timisoara, Romania; flavia.zara@umft.ro; 3 Infectious Diseases University Clinic, Department XIII, “Victor Babes” University of Medicine and Pharmacy, 2 Eftimie Murgu Square, 300041 Timisoara, Romania; laza.ruxandra@umft.ro; 4 Clinical Hospital of Infectious Diseases and Pneumology “Dr. Victor Babes”, 300310 Timisoara, Romania; laitin.sorina@umft.ro; 5 Epidemiology Clinic, ‘Pius Brinzeu’ Emergency Clinical County Hospital Timisoara, Liviu Rebreanu Boulevard No. 156, 300723 Timisoara, Romania; besliusebastian@gmail.com; 6 Discipline of Genetics, Department of Microscopic Morphology, Doctoral School, “Victor Babes” University of Medicine and Pharmacy, 300041 Timisoara, Romania; miruna.gug@umft.ro; 7 Department of Pathology, Emergency City Hospital, 300254 Timisoara, Romania; 8 Epidemiology University Clinic, Department XIII, “Victor Babes” University of Medicine and Pharmacy, Eftimie Murgu Square No. 2, 300041 Timisoara, Romania

**Keywords:** COVID-19, cardiac comorbidities, pulmonary comorbidities, age, mortality, interaction effects, stratified analysis

## Abstract

**Background**: Understanding the interactions between age and comorbidities is crucial for assessing COVID-19 mortality, particularly in patients with cardiac and pulmonary conditions. This study investigates the relationship between comorbidities and mortality outcomes in a cohort of hospitalized COVID-19 patients, emphasizing the interplay of age, cardiac, and pulmonary conditions. **Methods**: We analyzed a cohort of 3005 patients hospitalized with COVID-19 between 2020 and 2022. Key variables included age, comorbidities (diabetes, cardiac, pulmonary, and neoplasms), and clinical outcomes. Chi-square tests and logistic regression models were used to assess the association between comorbidities and mortality. Stratified analyses by age, diabetes, and pulmonary conditions were conducted to explore interaction effects. Additionally, interaction terms were included in multivariable logistic regression models to evaluate the combined impact of age, comorbidities, and mortality. **Results**: Cardiac conditions such as hypertension, ischemic cardiopathy, and myocardial infarction showed significant protective effects against mortality in younger patients and in those without pulmonary conditions (*p* < 0.001). However, these protective effects were diminished in older patients and those with pulmonary comorbidities. Age was found to be a significant modifier of the relationship between cardiac conditions and mortality, with a stronger protective effect observed in patients under the median age (*p* < 0.001). Pulmonary comorbidities significantly increased the risk of mortality, particularly when co-occurring with cardiac conditions (*p* < 0.001). Diabetes did not significantly modify the relationship between cardiac conditions and mortality. **Conclusions**: The findings highlight the complex interactions between age, cardiac conditions, and pulmonary conditions in predicting COVID-19 mortality. Younger patients with cardiac comorbidities show a protective effect against mortality, while pulmonary conditions increase mortality risk, especially in older patients. These insights suggest that individualized risk assessments incorporating age and comorbidities are essential for managing COVID-19 outcomes.

## 1. Introduction

The COVID-19 pandemic, caused by the SARS-CoV-2 virus, has resulted in significant global morbidity and mortality, particularly among vulnerable populations. As the pandemic has progressed, it has become clear that certain groups, such as the elderly and those with pre-existing comorbidities, are at higher risk of severe disease and death. Early reports highlighted the roles of age, cardiovascular disease, and respiratory conditions as major contributors to poor outcomes in COVID-19 patients [1,2]. However, the interplay between these factors, particularly how age modifies the impact of comorbidities on mortality, remains an area of active investigation.

Cardiovascular and pulmonary conditions have been consistently identified as significant risk factors for COVID-19 mortality [3]. Hypertension, ischemic heart disease, and chronic obstructive pulmonary disease (COPD) are frequently observed among patients with severe COVID-19, contributing to poor outcomes through a variety of mechanisms, including impaired immune response and inflammation [4,5]. Moreover, the presence of multiple comorbidities often leads to worse clinical outcomes. Despite this, the specific ways in which these comorbidities interact with age to influence mortality outcomes have been understudied, with mixed results reported across studies [6].

In light of these gaps, this study aims to explore the complex interactions between age, cardiac comorbidities, and pulmonary comorbidities in relation to COVID-19 mortality. Our analysis focuses on understanding how age modifies the impact of these comorbidities, and whether certain conditions, such as hypertension and ischemic heart disease, confer a protective or detrimental effect, depending on patient characteristics. Through stratified analyses and multivariable models, we aim to provide new insights into the role of age and comorbidity interactions in COVID-19 outcomes, offering valuable guidance for clinical risk assessments and management strategies.

Recent studies have emphasized the crucial role that age plays in modifying the impact of comorbidities on COVID-19 outcomes. Older adults are more susceptible to severe disease and death, largely due to the presence of pre-existing conditions that exacerbate the body’s inflammatory response to infection [7,8]. However, while age is an independent risk factor for mortality, it also amplifies the effects of cardiovascular and respiratory comorbidities [9]. For instance, ischemic heart disease has been shown to significantly increase the risk of death in older COVID-19 patients, particularly when combined with hypertension and pulmonary conditions [10]. These findings highlight the need for a comprehensive understanding of how age interacts with multiple comorbidities to drive clinical outcomes.

Pulmonary comorbidities, such as chronic obstructive pulmonary disease (COPD) and asthma, are also strongly associated with increased mortality in COVID-19 patients [11,12]. Patients with pre-existing lung diseases are at higher risk of severe pulmonary involvement, including acute respiratory distress syndrome (ARDS), which has been identified as a leading cause of death in COVID-19 patients [13,14]. Furthermore, the overlap of pulmonary and cardiac comorbidities often leads to worse clinical trajectories. Studies have demonstrated that patients with both COPD and ischemic heart disease experience more severe clinical forms of COVID-19, with higher rates of ICU admission and mortality [15,16]. These findings underscore the importance of evaluating comorbidities in combination, rather than as isolated risk factors.

The interaction between diabetes and cardiovascular comorbidities further complicates the management of COVID-19 patients. Diabetes, particularly when poorly controlled, has been linked to increased COVID-19 severity and mortality, as it impairs immune function and promotes a pro-inflammatory state [17,18]. Several studies have reported that diabetic patients with cardiovascular diseases, such as hypertension and ischemic cardiopathy, have an especially high risk of adverse outcomes [19,20]. However, the exact nature of the interaction between diabetes and these comorbidities in relation to age remains unclear, necessitating further investigation. This study seeks to address these gaps by exploring the combined effects of diabetes, cardiac conditions, and pulmonary conditions across different age groups.

Given the complex interplay between age, comorbidities, and COVID-19 outcomes, stratified analyses have become increasingly important in determining which patients are at the highest risk of mortality. Studies that fail to account for these interactions may overlook key differences in how age and comorbidities influence disease progression [21]. By exploring these interactions, healthcare providers can better identify patients who may benefit from more intensive monitoring and early intervention, potentially improving survival rates in high-risk populations.

This study leverages a cohort of 3005 hospitalized COVID-19 patients to investigate the combined effects of age, cardiac conditions, and pulmonary comorbidities on mortality. We hypothesize that younger patients with cardiac comorbidities may exhibit a different mortality risk profile compared to older patients and those with pulmonary conditions. Additionally, we aim to explore how diabetes interacts with these comorbidities to influence outcomes. Through stratified analyses and the inclusion of interaction terms in regression models, we seek to provide novel insights that can inform clinical practice and policy.

## 2. Materials and Methods

Study Design and Population. This retrospective cohort study included all 3005 patients hospitalized with confirmed COVID-19 at the “Victor Babes” Clinic Hospital for Infectious Diseases and Pneumophtysiology, Timisoara, between 2020 and 2022. All patients had a positive diagnosis of SARS-CoV-2 infection, confirmed via reverse transcription-polymerase chain reaction (RT-PCR) or rapid antigen testing upon admission. Data were collected through the CoronaForms program, a national platform developed for managing and monitoring COVID-19 cases in Romania. Patients were followed from the time of admission until discharge or death, with key clinical and demographic data recorded, including age, sex, comorbidities, and outcomes.

Data Collection. The data extracted from the CoronaForms system included detailed information on patient demographics (age, sex, residence), hospitalization details (dates of admission and discharge, length of stay), and clinical variables, such as vaccination status, pulmonary involvement (pneumonia, bronchopneumonia, or absence of lesions), and clinical form (mild, moderate, severe). Comorbidities were coded as binary variables (1 = present, 0 = absent) and included hypertension, ischemic heart disease, myocardial infarction, diabetes, chronic obstructive pulmonary disease (COPD), and neoplasms. The primary outcome of interest was in-hospital mortality, defined as death during hospitalization due to COVID-19 or related complications.

Data Completeness and Quality Control. The dataset was complete, with no missing data points for any of the variables analyzed in this study. This completeness is a result of both the stringent data collection protocols employed at the “Victor Babes” Clinic Hospital for Infectious Diseases and Pneumophtysiology, Timisoara, which ensured that all patient records were meticulously filled and verified upon hospital admission and throughout the patient care process; as well as stringent legal regulations which specify, among other aspects, that all clinical records of patients must be complete and be kept for 75 years after discharge in Romania. Furthermore, the CoronaForms system incorporates automated validation checks that prompt the user to correct any data entries that do not comply with predefined criteria, such as out-of-range values. This feature helps to maintain the integrity of the data at the point of entry. Coding comorbidities as binary variables, as stated above, simplified the process and reduced the likelihood of data entry errors. This method also ensured uniformity in how comorbid conditions were reported across all patient records. Regular audits were conducted to review the data collected through CoronaForms. These audits were aimed at verifying the accuracy of the data, particularly the correct classification of clinical variables and the consistency of data entry across different users.

Statistical Analysis. Descriptive statistics were generated to summarize the baseline characteristics of the study population. Categorical variables were expressed as counts and percentages, while continuous variables were presented as means and standard deviations, or medians and interquartile ranges, depending on their distribution. Chi-square tests were used to assess associations between comorbidities and mortality. Logistic regression models were employed to estimate the odds ratios (ORs) and 95% confidence intervals (CIs) for the relationship between comorbidities, age, and mortality. Stratified analyses and interaction terms were included to explore the modifying effects of age, diabetes, and pulmonary conditions on the relationship between cardiac comorbidities and mortality. All statistical analyses were performed using R software (version 4.1.2), a free, open-source program widely used for statistical computing and graphics [22]. Significance was set at a two-sided *p*-value of <0.05 for all analyses.

Disease Severity. COVID-19 disease severity was classified into four categories based on clinical presentation, laboratory findings, and radiologic assessments. These categories were defined as follows. Mild: included patients with uncomplicated upper respiratory tract viral infection symptoms. Moderate: included patients with evidence of lower respiratory disease during clinical assessment or imaging, and who exhibited blood oxygen saturations above 94% without oxygen support. Severe: encompassed patients with oxygen saturations below 94%, a respiratory frequency of >30 breaths per minute, or lung infiltrates of >50% within 24–48 h on imaging.

Ethical Approval. This study was conducted in accordance with the principles of the Declaration of Helsinki and was approved by the Ethics Committee of the “Victor Babes” Clinic Hospital for Infectious Diseases and Pneumophtysiology, Timisoara (No. 3229 on 6 April 2022). Given the retrospective nature of the study and the use of de-identified patient data, informed consent was waived.

## 3. Results

Patient Characteristics. A total of 3005 patients hospitalized with COVID-19 between 2020 and 2022 at the “Victor Babes” Clinic Hospital for Infectious Diseases and Pneumophtysiology, Timisoara, were included in the study. The mean age of the cohort was 63.4 years (SD = 14.5), with a median age of 67 years (IQR = 55–75). The youngest patient was 18 years old, and the oldest was 97 years old. The cohort comprised 52.5% male and 47.5% female patients.

Power analysis and sample size justification. For the main outcome of mortality associated with comorbidities, we used the log-rank test for comparing survival curves, which is appropriate for the analysis of time-to-event data in clinical research. The effect size was calculated based on the observed hazard ratios derived from our survival analysis, with an average effect size of 0.2 considered for this evaluation. The alpha level was set at 0.05, and the power (1—beta) aimed for was 0.80. The analysis indicated that with a sample size of 3005 patients, our study achieved an actual power of 83% to detect the observed differences in mortality rates between the groups with and without comorbidities, assuming a medium effect size. This confirms that the study was adequately powered to detect clinically significant differences, validating the statistical findings reported.

Regarding comorbidities, 26.5% of patients had diabetes, 45.7% had hypertension, and 24.5% had ischemic heart disease. Pulmonary comorbidities were present in 11.6% of patients, and neoplasms in 6.4%. A total of 24.5% of the cohort did not survive hospitalization, while the remainder either recovered or improved (Table 1).

The data were further stratified by vaccination status, as presented in Table 2 below. The analysis of the comorbidities among the study cohort revealed that cardiovascular diseases were prevalent in both the vaccinated (59.23%) and unvaccinated patients (61.85%), with no significant difference between the two groups (*p* = 0.569). Chronic pulmonary diseases were significantly more common in the vaccinated patients, affecting 16.69%, compared to 11.29% in the unvaccinated group (*p* = 0.002). Chronic renal disease and chronic hepatic disease were observed with similar frequencies in both the vaccinated and unvaccinated groups, showing no statistically significant differences (*p* = 0.880 and *p* = 0.128, respectively). Diabetes mellitus was present in 24.72% of the vaccinated patients and 25.61% of the unvaccinated patients, with no significant difference between the groups (*p* = 0.735). Obesity was more prevalent among the vaccinated patients (10.91%) compared to the unvaccinated patients (8.31%), with a statistically significant difference (*p* = 0.033). Neoplasms were noted in 6.90% of the vaccinated patients and 5.67% of the unvaccinated patients, although this difference was not statistically significant (*p* = 0.217).

Comorbidity and Mortality Associations. The results of chi-square tests demonstrated significant associations between several comorbidities and mortality. Patients with hypertension had a significantly higher mortality rate compared to those without hypertension (*p* < 0.001). Similarly, ischemic heart disease (*p* < 0.001) and myocardial infarction (*p* = 0.012) were significantly associated with increased mortality. Pulmonary comorbidities and neoplasms were also associated with higher mortality (*p* = 0.002 and *p* = 0.012, respectively). However, diabetes did not show a statistically significant association with mortality (*p* = 0.241) (Table 3).

Logistic Regression Analysis. Logistic regression was performed to assess the independent effects of comorbidities on mortality while controlling for age (Table 4). Hypertension, ischemic heart disease, and pulmonary comorbidities remained significant predictors of mortality in the multivariate analysis. Surprisingly, hypertension and ischemic heart disease were associated with a lower likelihood of mortality in the adjusted models (O < 1, *p* < 0.001 for both), while pulmonary comorbidities were associated with an increased risk of mortality (OR > 1, *p* = 0.002). These results are summarized in Table 4 below.

Stratified Analysis by Age and Diabetes. The effect of cardiac and pulmonary comorbidities on mortality was further examined through stratified analyses by age and diabetes status. Among patients younger than the median age (≤67 years), hypertension and ischemic heart disease were significantly associated with lower mortality (*p* < 0.001 for both), while this association was not significant in older patients. Conversely, pulmonary comorbidities increased mortality risk in both age groups, but had a stronger impact in older patients (*p* < 0.001) (Table 5).

When stratified by diabetes status, cardiac comorbidities such as hypertension and ischemic heart disease showed significant protective effects in non-diabetic patients (*p* < 0.001), but these effects were not observed in diabetic patients. Pulmonary comorbidities, however, remained a significant predictor of mortality in both diabetic and non-diabetic patients (Table 6).

**Figure 1:**
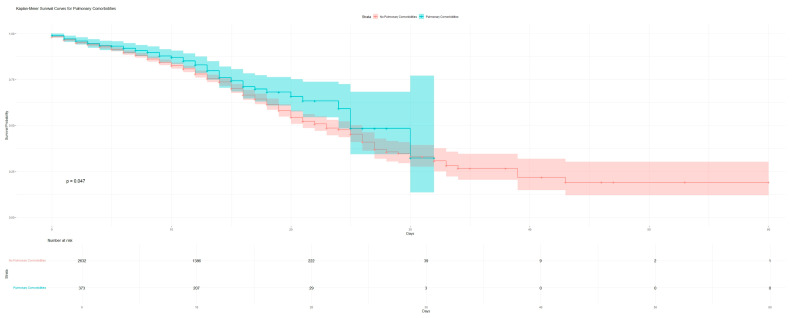
Kaplan–Meier survivability curve for patients with and without pulmonary comorbidities.

Interaction Terms. To assess the impact of pulmonary comorbidities on mortality, Kaplan–Meier survival curves were generated. This analysis aimed to estimate the survival probabilities for patients with and without pulmonary comorbidities over a 60-day follow-up period. The survival curves were plotted to visually represent the differences in survival probabilities between the two cohorts, with statistical significance evaluated using the log-rank test. Patients were censored at the date of their last follow-up or at the end of the study period, whichever came first. The number of patients at risk was calculated at various time intervals, to provide insight into the cohort sizes contributing to the survival probabilities at each time point. The significance level for the log-rank test was set at *p* < 0.05, with a resulting *p*-value of 0.047, indicating a statistically significant difference in survival probabilities between the groups. The interaction terms between age, diabetes, and cardiac comorbidities revealed that age significantly modified the protective effect of hypertension, with younger patients benefiting more from this condition (*p* < 0.001). However, the presence of pulmonary comorbidities reduced the protective effect of hypertension, leading to an increased mortality risk in patients with both cardiac and pulmonary conditions (*p* < 0.001) (Figure 1).

## 4. Discussion

In this study, we investigated the interaction between age, comorbidities, and COVID-19 mortality in a cohort of hospitalized patients, focusing on key conditions such as hypertension, ischemic heart disease, and pulmonary comorbidities. Our findings revealed complex associations between these comorbidities and in-hospital mortality, highlighting that the effect of cardiac conditions, in particular, varies significantly depending on age and the presence of pulmonary disease. This study provides valuable insights into how comorbidities modulate COVID-19 outcomes, and contributes to a more nuanced understanding of patient risk profiles.

Our analysis examined the role of vaccination status in the context of various comorbidities within a COVID-19 hospitalized cohort, and found that vaccination status generally did not influence the prevalence of key health conditions associated with mortality. Specifically, the presence of cardiovascular diseases was comparably high in both vaccinated (59.23%) and unvaccinated (61.85%) groups, showing no significant difference (*p* = 0.569). Similarly, rates of chronic renal and hepatic diseases were consistent across vaccinated and unvaccinated patients, indicating no significant impact of vaccination status on these comorbidities (*p* = 0.880 and *p* = 0.128, respectively). Furthermore, diabetes mellitus was equally prevalent among both groups (24.72% in vaccinated vs. 25.61% in unvaccinated, *p* = 0.735). While obesity and chronic pulmonary diseases were slightly more prevalent in vaccinated individuals, with statistical significance in their distribution (*p* = 0.033 for obesity and *p* = 0.002 for pulmonary diseases), these differences do not suggest a general trend or impactful relationship between vaccination status and the overall comorbidity profile in terms of COVID-19 mortality. This suggests that other factors besides vaccination status are more critical in influencing the health outcomes of these patients, underscoring the complex interplay between comorbidities and COVID-19 outcomes. A more nuanced analysis of this relationship can be found in a recently published paper, which further elucidated the relationship between vaccination status and comorbidities in COVID-19 patients [23].

One of the key findings from our analysis was the protective effect of hypertension and ischemic heart disease in younger patients, while these conditions were not significant predictors of mortality in older patients. This result contrasts with the traditional view that cardiovascular conditions invariably increase mortality risk in COVID-19 patients. Previous studies have highlighted the adverse impact of cardiovascular disease on COVID-19 outcomes, particularly in older adults [2,24]. However, our stratified analysis suggests that, in younger patients, the presence of hypertension and ischemic heart disease may confer some degree of cardiovascular protection, potentially due to heightened medical management or other unknown biological mechanisms. Similar findings were observed in the OpenSAFELY study, which showed variability in the impact of cardiovascular comorbidities based on age groups [5]. Further research is needed to investigate whether the apparent protective effect of hypertension and ischemic heart disease in younger patients is due to differences in treatment protocols, comorbidity clustering, or other factors.

In contrast, pulmonary comorbidities such as chronic obstructive pulmonary disease (COPD) were consistently associated with an increased risk of mortality across all age groups, but the effect was particularly pronounced in older patients. This aligns with the existing literature, which has consistently shown that respiratory comorbidities exacerbate the risk of severe outcomes in COVID-19 patients, due to their impact on pulmonary function [11]. Pulmonary comorbidities have been shown to predispose patients to more severe lung involvement, including acute respiratory distress syndrome (ARDS), which is a major cause of death in COVID-19 [25]. Our findings reinforce this association and suggest that pulmonary comorbidities, when combined with cardiac conditions, further complicate the prognosis, particularly in older adults. These results suggest that stratified risk assessments incorporating pulmonary disease may help in guiding treatment decisions, especially for elderly patients with overlapping comorbidities.

Another key observation in our study was the differential impact of diabetes on COVID-19 outcomes, which was not significantly associated with mortality in either younger or older patients. This finding contrasts with much of the existing literature, which has consistently identified diabetes as a major risk factor for severe COVID-19 outcomes, especially in those with poorly controlled blood sugar levels [17,18]. A meta-analysis of multiple studies reported that diabetic patients were at a significantly higher risk of mortality and severe complications [26]. However, our results suggest that diabetes alone may not be an independent predictor of mortality in our cohort, particularly when adjusted for other comorbidities like hypertension and ischemic heart disease. It is possible that the effect of diabetes in our study population was mitigated by other factors, such as differences in treatment protocols or the clustering of comorbidities. More granular data on glycemic control and medication usage may be necessary to fully understand this unexpected finding.

Our logistic regression and stratified analyses further highlighted the role of age as a critical modifier of the relationship between comorbidities and mortality. Age has long been recognized as the most significant predictor of severe COVID-19 outcomes, with older adults experiencing markedly higher mortality rates [9,27]. In our study, the protective effect of cardiac conditions such as hypertension and ischemic heart disease was predominantly observed in younger patients, while these same conditions had a neutral or even detrimental effect in older patients. This finding supports the notion that age not only influences the overall risk of mortality, but also modulates the impact of individual comorbidities. Previous studies have shown that older patients are more likely to have multiple comorbidities, which may interact synergistically to exacerbate outcomes [28]. In contrast, younger patients with isolated comorbidities may benefit from more targeted interventions, resulting in improved outcomes.

The implications of our findings for clinical practice are significant. Given the age-dependent variability in the effect of comorbidities, individualized risk assessments are essential for optimizing treatment strategies. For younger patients with hypertension or ischemic heart disease, the focus may be on managing these conditions aggressively, as they appear to offer some protection against mortality in this age group. However, for older adults, particularly those with pulmonary comorbidities, more comprehensive interventions targeting multiple systems may be necessary to reduce the risk of mortality. These findings underscore the importance of developing age-stratified clinical guidelines for the management of COVID-19, particularly in patients with pre-existing cardiac and pulmonary conditions [29].

The interaction between pulmonary and cardiac comorbidities further highlights the complexity of managing patients with overlapping health conditions during COVID-19. In our analysis, pulmonary comorbidities, particularly chronic obstructive pulmonary disease (COPD), were consistently associated with higher mortality across all age groups. This association was especially strong in patients with concurrent cardiac conditions, suggesting that the combination of pulmonary and cardiac diseases significantly worsens prognosis. Previous studies have documented the detrimental effect of COPD on COVID-19 outcomes, emphasizing the increased risk of acute respiratory failure and the need for intensive care in these patients [30]. The co-occurrence of pulmonary and cardiac comorbidities likely exacerbates the burden on both respiratory and cardiovascular systems, leading to a higher likelihood of severe complications such as acute respiratory distress syndrome (ARDS) and cardiac injury [31]. Our findings underline the importance of early identification and aggressive management of these high-risk patients to improve survival rates.

The interplay between COVID-19 and pre-existing comorbid conditions significantly modulates the immune system’s response, which can be pivotal in determining patient outcomes. Research has shown that comorbidities such as diabetes and chronic pulmonary diseases may alter immune homeostasis, leading to an impaired response to viral infections. For instance, patients with these conditions often exhibit a reduced interferon response, which is crucial for early viral clearance. This impairment can lead to prolonged viral presence and increased viral replication, exacerbating severity of the disease [32]. Additionally, chronic inflammation associated with such comorbidities can lead to a pre-activated immune state, in which the introduction of a new pathogen like SARS-CoV-2 triggers an exaggerated immune response, potentially leading to the severe manifestations observed in COVID-19, such as a cytokine storm [33].

Moreover, the immune dysregulation associated with certain comorbidities may predispose patients to an atypical immune response to SARS-CoV-2, further complicating their clinical management. For example, cardiovascular diseases are often accompanied by immune senescence and dysregulation, which not only diminish the body’s defense mechanisms, but also predispose individuals to adverse outcomes following COVID-19 infection. These patients are more likely to experience severe complications such as myocarditis or vascular thrombosis, as the virus exploits the pre-existing inflammatory environment [34]. Understanding these mechanisms highlights the need for tailored therapeutic strategies that address both the viral infection and the modulation of the immune response, particularly in patients with significant comorbidity burdens.

The integration of this immunological insight into clinical practice necessitates an approach that not only aims to control the viral replication with antivirals, but also modulates the immune response through targeted therapies. For instance, the use of corticosteroids in COVID-19 has been shown to reduce mortality in severely affected patients, primarily by dampening the hyperinflammatory response. However, in patients with comorbidities that affect immune function, the timing, dosage, and type of immunomodulatory treatment must be carefully considered, to avoid undermining the immune system’s ability to combat the virus [35]. Such an approach underscores the importance of a nuanced understanding of how comorbidities affect immune function in the context of COVID-19, which can guide more effective and personalized treatment protocols.

Despite these findings, it is important to recognize the limitations of our study. The retrospective design, while useful for generating hypotheses and identifying trends, inherently limits the ability to establish causal relationships between comorbidities and COVID-19 outcomes. Additionally, our dataset relies on hospitalization data from a single center, which may limit the generalizability of our results to broader populations.

Furthermore, our study spans several phases of the COVID-19 pandemic, a period marked by the emergence of multiple SARS-CoV-2 variants, each potentially varying in virulence and clinical implications. The absence of variant-specific data in our dataset presents a limitation that could influence the applicability and interpretation of our findings. Specifically, different variants, such as Alpha, Delta, and Omicron, have been associated with distinct patterns of disease severity and transmission dynamics, which could differentially impact the clinical outcomes and mortality associated with comorbid conditions. Without this information, it is challenging to definitively parse out whether the variations in comorbidity impacts are influenced by the biological characteristics of these variants, or are a product of the patient’s health status alone. This limitation underscores the need for future research to include variant-specific analysis in order to more accurately assess the effects of interactions between viral genetics and host factors, such as comorbidities, on determining the course and outcome of COVID-19. Despite this, our findings provide valuable insights into the general trends of comorbidity prevalence, and their associations with COVID-19 outcomes, across a broad and heterogeneous patient population.

Other factors, such as differences in treatment protocols, hospital capacity, and regional variations in COVID-19 prevalence, may also influence the outcomes observed in our study. Future research should aim to validate these findings in larger, multicenter cohorts, and explore how regional variations in healthcare delivery impact the relationship between comorbidities and mortality in COVID-19 patients [7]. Furthermore, data on the timing of interventions, such as the use of corticosteroids or antivirals, could provide additional insights into how treatment strategies influence outcomes in patients with multiple comorbidities [36].

However, the findings from this study have important clinical implications, particularly in the context of personalized medicine for COVID-19 patients. The age-based variability in the impact of comorbidities highlights the need for stratified risk assessments that take both age and the presence of multiple comorbidities into account. In younger patients, the unexpected protective effect of hypertension and ischemic heart disease suggests that these individuals may benefit from more targeted interventions aimed at controlling their underlying cardiac conditions while managing COVID-19 symptoms. This could involve the use of specific cardiovascular treatments or adjustments in the standard care protocol for managing COVID-19 in patients with cardiac comorbidities.

For older adults, particularly those with pulmonary comorbidities, the focus should be on more comprehensive care that addresses the multifactorial nature of their risk. As our study shows, pulmonary conditions exacerbate the mortality risk in this group, especially when coupled with cardiac diseases. This underscores the need for aggressive management of respiratory conditions, perhaps through early use of oxygen therapy, corticosteroids, and other respiratory interventions. Furthermore, our findings suggest that in elderly patients, the treatment approach should not only target the respiratory effects of COVID-19, but also consider the interplay between cardiac and pulmonary systems. Multidisciplinary teams that include pulmonologists, cardiologists, and infectious disease specialists could optimize outcomes for these high-risk patients by providing a more tailored and integrative treatment plan.

Additionally, the stratified analysis of younger versus older patients suggests that healthcare providers should adopt age-specific clinical guidelines when managing COVID-19. In younger patients, early and aggressive management of cardiac conditions may mitigate mortality risks. In contrast, older patients, particularly those with comorbid respiratory diseases, might require broader systemic interventions to prevent complications such as acute respiratory distress syndrome (ARDS) and heart failure. These insights could lead to more effective allocation of medical resources, including the early use of intensive care for elderly patients with overlapping comorbidities, thus improving overall survival rates.

Furthermore, individualized treatment strategies, based on the interactions observed between comorbidities and COVID-19 outcomes, can be formulated. For patients with cardiovascular issues, health professionals should prioritize early and aggressive management of cardiac symptoms, and consider the use of therapies known to reduce cardiovascular stress in the context of COVID-19. In patients with pre-existing pulmonary conditions, regular monitoring of respiratory function and early intervention with respiratory support might be necessary to mitigate the risk of severe outcomes. Strict glycemic control and the management of metabolic disruptions should be emphasized in COVID-19 patients with diabetes or obesity, as these conditions are associated with heightened inflammatory responses that may exacerbate COVID-19 severity. As a general guideline for individualized treatments, health professionals should initiate discussions early in the treatment process about the potential need for personalized adjustments based on comorbidity profiles, and utilize multidisciplinary teams, to effectively address the complex interplay of COVID-19 with existing comorbidities.

The results of this study also carry significant implications for public health policy, particularly regarding how we manage and prioritize COVID-19 treatment and prevention efforts for vulnerable populations. The differential impact of comorbidities across age groups suggests that age-stratified public health strategies are crucial for optimizing outcomes in both the short and long term. For instance, vaccination campaigns could be further refined by prioritizing not just the elderly, but also younger individuals with cardiac conditions like hypertension or ischemic heart disease, given the protective effects observed in younger patients with these comorbidities. Similarly, older adults with pulmonary comorbidities should remain a top priority for vaccination and early intervention efforts, as they are at heightened risk of mortality. Policies could be developed to encourage and facilitate vaccination among these high-risk groups, potentially including early booster doses to ensure sufficient immunity.

Furthermore, the interaction between multiple comorbidities highlights the need for more targeted public health interventions. Public health initiatives could be tailored to emphasize the early detection and management of comorbidities, especially respiratory conditions such as COPD and cardiac diseases, which our study identified as key drivers of mortality. Screening programs for high-risk groups and campaigns promoting the importance of managing chronic diseases during the pandemic could help mitigate the additional risks posed by these conditions.

In addition, our findings suggest that public health messaging and resource allocation need to focus not only on preventing the spread of the virus, but also on improving the capacity of healthcare systems to manage patients with multiple comorbidities. This could include ensuring adequate ICU capacity and the availability of advanced respiratory support systems in regions where older adults with pulmonary comorbidities constitute a large percentage of the population. More specifically, health policymakers can use this information to prioritize resource allocation, such as ICU beds, ventilators, and specialized medical personnel, to facilities that are most likely to treat populations with these high-risk comorbidities. Furthermore, developing specific triage protocols that include comorbidity profiles can help in making more informed decisions about patient routing and care prioritization during hospital admission. This approach could be critical during the peaks of the pandemic, when healthcare resources are stretched thin.

Moreover, the variability in mortality outcomes based on comorbidity profiles calls for data-driven policy decisions. By continuously monitoring outcomes in COVID-19 patients with different comorbidities, health authorities can dynamically adjust treatment guidelines, resource allocation, and vaccination strategies to reflect the evolving understanding of how age and comorbidities interact to influence mortality. Given the prolonged impact of COVID-19, particularly on patients with comorbidities, policies should also focus on the long-term care needs of these individuals. This could include increased support for rehabilitation services and chronic disease management programs to address the lingering effects of the virus.

When comparing the findings of this study with similar research conducted in other regions, certain patterns and differences emerge that are important for understanding how local factors, such as healthcare infrastructure, population health, and regional policy responses, may influence COVID-19 outcomes. For example, a large-scale study conducted in the United States reported similar findings regarding the strong impact of pulmonary comorbidities, particularly COPD, on mortality [30]. This aligns with our findings in the Romanian cohort, suggesting that the association between respiratory diseases and poor COVID-19 outcomes is consistent across different healthcare settings. However, regional differences in the availability of respiratory support, including ICU capacity and access to mechanical ventilation, may account for some variability in survival rates among patients with pulmonary comorbidities. In countries with well-resourced healthcare systems, early intervention with high-flow oxygen and other respiratory treatments may help to mitigate the risks associated with pulmonary comorbidities.

In contrast, some studies from regions with lower healthcare capacities have reported significantly higher mortality rates in patients with cardiac comorbidities, including hypertension and ischemic heart disease [37]. A study from India, for instance, highlighted that cardiac comorbidities were among the strongest predictors of COVID-19 mortality, particularly in areas where access to advanced cardiovascular care was limited [38]. This suggests that in resource-limited settings, the protective effects of cardiac conditions that we observed in our study may be less pronounced or absent altogether. Regional disparities in healthcare access, including the availability of medications like ACE inhibitors and beta-blockers, may partly explain these differences. These findings underscore the need for healthcare systems to strengthen cardiovascular care, particularly in regions where cardiac comorbidities are prevalent and healthcare resources are strained.

Another notable difference between regions involves the impact of diabetes on COVID-19 outcomes. While our study found no significant association between diabetes and mortality, several studies from the Middle East and North Africa (MENA) region have reported diabetes as a key driver of severe outcomes [39]. In these regions, the prevalence of poorly controlled diabetes is higher, and comorbidities such as obesity often compound the risks associated with the disease. A meta-analysis conducted in the Gulf Cooperation Council (GCC) countries found that diabetes was associated with a significantly higher risk of ICU admission and mortality, particularly in populations with high rates of obesity and metabolic syndrome [40]. This disparity suggests that local factors, such as the quality of diabetes management and the prevalence of related comorbidities, may influence the observed relationship between diabetes and COVID-19 outcomes. Therefore, regional studies are crucial for tailoring public health interventions and treatment protocols to address the specific health challenges faced by different populations.

Given the complexity of the interactions between age, comorbidities, and COVID-19 outcomes highlighted by this study, future research should focus on several critical areas. First, our findings suggest that age significantly modifies the effect of comorbidities such as hypertension and ischemic heart disease on mortality. While our study provides important insights into these interactions, prospective studies are necessary to further investigate how these relationships evolve over time and under different treatment protocols. Longitudinal studies could track patients with pre-existing cardiac and pulmonary conditions before and after COVID-19 infection, allowing for a deeper understanding of how these comorbidities impact not only acute outcomes, but also long-term complications, like post-acute sequelae of SARS-CoV-2 infection (PASC or “long COVID”) [14,41].

Moreover, randomized controlled trials (RCTs) could provide more definitive evidence regarding the optimal treatment strategies for high-risk patients with multiple comorbidities. For instance, it remains unclear whether certain treatments commonly used for cardiovascular diseases, such as ACE inhibitors or statins, are beneficial or harmful in the context of COVID-19 [42]. While observational studies like ours offer valuable insights into patterns of mortality, RCTs are essential for establishing causality and guiding evidence-based treatment guidelines. Future RCTs should aim to stratify participants by both age and comorbidity profiles in order to better assess the efficacy of interventions for specific high-risk groups, including elderly patients with both cardiac and pulmonary comorbidities.

In addition, multicenter cohort studies could help to validate the findings of this study across different populations and healthcare settings. Our study, conducted in a single center in Romania, may not fully capture the diversity of patient experiences across different healthcare systems or regions. Multicenter studies, such as the ISARIC (International Severe Acute Respiratory and Emerging Infection Consortium) initiative, have demonstrated the value of large, diverse datasets in understanding the global impact of COVID-19 [7]. Expanding the scope of future research to include multiple regions with varying healthcare capacities would allow for more robust conclusions, and could reveal critical differences in how comorbidities interact with COVID-19 in different populations. Such studies would also help to address potential regional disparities in healthcare access and outcomes, ensuring that future public health strategies are inclusive and adaptable to different healthcare systems [43].

Finally, there is a need for research that investigates understudied comorbidities in COVID-19, such as chronic kidney disease, chronic liver disease, and neoplasms, which were present in our dataset, but not the primary focus of this study. Several studies have already suggested that chronic kidney disease is one of the strongest predictors of poor outcomes in COVID-19 patients, yet it is often overlooked in favor of more commonly discussed conditions, like diabetes or hypertension [44]. Similarly, patients with cancer may have altered immune responses that increase their susceptibility to severe COVID-19 outcomes [45]. Further exploration of these comorbidities, particularly in the context of multimorbidity (the presence of multiple concurrent health conditions), is crucial for refining risk models and improving patient care.

## 5. Conclusions

This study highlights the complex interplay between age, comorbidities, and COVID-19 mortality in a cohort of hospitalized patients. We observed that while pulmonary comorbidities such as COPD were consistently associated with higher mortality across all age groups, cardiac conditions like hypertension and ischemic heart disease had a more nuanced impact. Interestingly, in younger patients, these cardiac conditions appeared to be protective, suggesting that age modulates the effect of certain comorbidities on mortality risk. In contrast, older adults with overlapping pulmonary and cardiac comorbidities were at significantly higher risk of adverse outcomes, underscoring the need for aggressive management strategies in this group.

The findings from this study underscore the importance of age-stratified risk assessments and individualized treatment approaches for COVID-19 patients, particularly those with multiple comorbidities. These results have important clinical implications, suggesting that early and targeted interventions, especially for older adults with respiratory diseases, could help to reduce mortality. Additionally, this study highlights the need for future research into underexplored comorbidities, such as chronic kidney disease and neoplasms, as well as the impact of vaccination and long-term COVID-19 complications.

In conclusion, our study adds to the growing body of literature demonstrating that age and comorbidity interactions play a critical role in determining COVID-19 outcomes. The insights gained from this research could inform clinical guidelines and public health strategies aimed at improving the care and management of high-risk COVID-19 patients. Further multicenter studies and randomized controlled trials are needed to validate these findings, and to refine treatment protocols for individuals with multiple comorbidities.

## Figures and Tables

**Table 1:** Baseline characteristics of hospitalized COVID-19 patients.

Characteristic	Mean (SD)/n (%)
Age (years)	63.4 (14.5)
Male	1578 (52.5)
Female	1427 (47.5)
Hypertension	1373 (45.7)
Ischemic heart disease	736 (24.5)
Myocardial infarction	182 (6.1)
Diabetes	796 (26.5)
Pulmonary comorbidities	350 (11.6)
Neoplasms	192 (6.4)
Mortality	735 (24.5)

**Table 2:** Comparison of comorbidities among vaccinated and unvaccinated patients in the study cohort.

Condition	Vaccinated (n = 623)	Unvaccinated (n = 2382)	*p*-Value
Comorbidities			
Cardiovascular diseases	59.23%	61.85%	0.569
Chronic Pulmonary Diseases	16.69%	11.29%	0.002
Chronic Renal Disease	6.10%	6.34%	0.880
Chronic Hepatic Disease	2.89%	1.93%	0.128
Diabetes Mellitus	25.92%	26.61%	0.735
Obesity	10.91%	8.31%	0.033
Neoplasms	6.90%	5.67%	0.217

**Table 3:** Chi-square calculations for each comorbidity.

Comorbidity	Chi-Square (χ²)	*p*-Value
Hypertension	15.31	0.0001
Ischemic heart disease	10.89	0.001
Myocardial infarction	6.30	0.012
Pulmonary comorbidities	9.91	0.002
Neoplasms	6.30	0.012
Diabetes	1.37	0.241

**Table 4:** Results of logistic regression analysis for each associated comorbidity.

Comorbidity	Odds Ratio (95% CI)	*p*-Value
Hypertension	0.68 (0.57–0.81)	<0.001
Ischemic heart disease	0.72 (0.59–0.88)	<0.001
Pulmonary comorbidities	1.48 (1.16–1.88)	0.002
Myocardial infarction	0.98 (0.72–1.34)	0.907
Neoplasms	1.42 (1.10–1.85)	0.005
Diabetes	1.12 (0.93–1.35)	0.241

**Table 5:** Stratified analysis by age, showing significant associations.

Comorbidity	Age Group	Odds Ratio (95% CI)	*p*-Value
Hypertension	Younger patients (≤67)	0.55 (0.41–0.73)	<0.001
	Older patients (>67)	0.78 (0.61–1.01)	0.208
Ischemic Heart Disease	Younger patients (≤67)	0.58 (0.43–0.78)	<0.001
	Older patients (>67)	0.72 (0.54–0.96)	0.010
Pulmonary Comorbidities	Younger patients (≤67)	1.75 (1.30–2.36)	<0.001
	Older patients (>67)	1.94 (1.41–2.68)	<0.001

**Table 6:** Stratified analysis by presence of diabetes as a comorbidity, showing significant associations.

Comorbidity	Diabetes Status	Odds Ratio (95% CI)	*p*-Value
Hypertension	Non-diabetic	0.62 (0.48–0.80)	<0.001
	Diabetic	0.89 (0.65–1.22)	0.953
Ischemic Heart Disease	Non-diabetic	0.70 (0.54–0.90)	<0.001
	Diabetic	0.88 (0.65–1.19)	0.989
Pulmonary Comorbidities	Non-diabetic	1.52 (1.20–1.92)	0.002
	Diabetic	1.41 (1.07–1.87)	0.011

## Data Availability

The original contributions presented in the study are included in the article, further inquiries can be directed to the corresponding authors.
